# A Treatise on Simple and Compound Ophthalmic Lenses

**Published:** 1887-03

**Authors:** 


**A Treatise on Simple and Compound Ophthalmic Lenses,** their Refraction and Diapetric Formulæ, including tables of Crossed Cylinders and their Sphero-Cylindrical Equivalents. By Chas. F. Prentice. New York: James Prentice & Son, opticians, 178 Broadway.

The firm with which the author of this book has been connected have long been known as skillful and trustworthy practical opticians. This book gives credence that Mr. Chas. Prentice is both theoretically and practically an accomplished optician. The book will be found most serviceable by opthalmologists as well as all opticians who attempt to make lenses in accordance with instructions. The demand for a work of this kind is necessarily limited, but the price has nevertheless been fixed at the small sum of $1.50.

